# The medial femoral wall can play a more important role in unstable intertrochanteric fractures compared with lateral femoral wall: a biomechanical study

**DOI:** 10.1186/s13018-017-0673-1

**Published:** 2017-12-28

**Authors:** Boyuan Nie, Xueying Chen, Jing Li, Dou Wu, Qiang Liu

**Affiliations:** 1 Department of Orthopedic Surgery, Dayi Hospital of Shanxi Medical University, NO.99 Longcheng Street, Taiyuan, Shanxi 030032 People’s Republic of China; 2 Biological Material R&D Center, China Institute for Radiation Protection, NO.102 Xuefu Street, Taiyuan, Shanxi 030006 People’s Republic of China

**Keywords:** Medial wall, Lateral wall, Femoral intertrochanteric region, Proximal femoral nail antirotation, Biomechanical testing, Cadaver femur

## Abstract

**Background:**

The major objective of the present study is to investigate the differences in the load and strain changes in the intertrochanteric region of human cadaveric femora between the loss of medial or lateral wall and after treatment with proximal femoral nail antirotation (PFNA).

**Methods:**

After measuring the geometry of the proximal femur region and modeling the medial or lateral wall defect femoral models, six pairs of freshly frozen human femora were randomly assigned in the medial or lateral wall group. According to a single-leg stance model, an axial loading was applied, and the strain distribution was measured before and after PFNA implantation. The strains of each specimen were recorded at load levels of 350, 700, and 1800 N and the failure load. Paired *t* test was performed to assess the differences between two groups.

**Results:**

The failure mode of almost all defect model femora was consistent with that of the simulated type of intertrochanteric fractures. After the PFNA implantation, the failure mode of almost all stabilized femora was caused by new lateral wall fractures. The failure load of the lateral wall group for defect model femora was significantly higher than that of the medial wall group (*p* < 0.001). However, the difference disappeared after the PFNA was implanted (*p* = 0.990). The axial stiffness in all defect model femora showed the same results (*p* < 0.001). After the PFNA implantation, the axial stiffness of the lateral wall group remained higher than that of the medial wall group (*p* = 0.001). However, the axial stiffness of the lateral wall group showed that the femora removed from the lateral wall were higher than the PFNA-stabilized femora (*p* = 0.020). For the axial strain in the anterior wall after the PFNA implantation, the strain of the lateral wall group was significantly lower than that of the medial group (*p* = 0.003). Nevertheless, for the axial strain of the posterior wall after the PFNA implantation, the strain of the medial wall group was significantly lower than that of the lateral group (*p* < 0.001).

**Conclusions:**

In summary, this study demonstrated that PFNA is an effective intramedullary fixation system for treating unstable intertrochanteric fractures. Compared with the lateral wall, the medial femoral wall is a more important part in the intertrochanteric region. We suggest that in treating intertrochanteric femoral fractures with medial wall fractures, the medial wall fragment should be reset and fixed as much as possible.

## Background

Intertrochanteric fractures are common and result in considerable mortality and morbidity, which cause a great financial burden to society. At present, except for comorbidities that place patients at unacceptable risk from anesthesia, surgical procedure, or both, surgical treatment of intertrochanteric hip fractures is usually reserved [1]. Fixation of intertrochanteric fractures is commonly performed using either an extramedullary sliding hip screw-plate construct or an intramedullary nail with a cephalomedullary screw [2].

Irrespective of which implant is chosen, the stability of an intertrochanteric fracture is important for treatment guidance and prognosis evaluation. The loss of integrity of the medial or lateral wall of an intertrochanteric region has been suggested as the most important cause of instability in intertrochanteric fractures. The medial wall of the femoral intertrochanteric region includes the medial cortex and medial calcar located in its deep side. The defect in the intertrochanteric medial wall has been proven to be due to the hinge, which postoperatively causes coxa vara and proximal femoral shortening after intertrochanteric fractures occur. The lateral femoral wall was defined for the first time in 2004 by Gotfried [3] as a proximal extension of the femoral shaft, whereas Palm [4] defined it as the lateral femoral cortex distal to the vastus ridge. In recent clinical studies, the lateral femoral wall has been recognized as an important predictor of stability in intertrochanteric fractures [3–6].

The main objective of the present study is to investigate the load and strain changes in the intertrochanteric region between the removed group from the medial wall and that from the lateral wall before and after having been treated using proximal femoral nail antirotation (PFNA) for human cadaveric femora.

## Methods

### Specimens

Six pairs of freshly frozen human cadaveric femora (*n* = 12, six males) with a mean age of 77.17 ± 4.36 years (range 70–82 years) were used in this study. All specimens were obtained from the Medical Tissue Bank of Shanxi Province in China. After dissection of the body, all specimens were stored at − 20 °C in doubly sealed plastic bags. X-rays were performed to ensure that the specimens did not have anatomical deformities or evidence of prior pathology, fracture, arthritis, or surgery. The geometry of the proximal femur region (head diameter, neck length, neck-shaft angle, and anteversion angle) was measured. Dual-energy X-ray absorptiometry (Hologic QDR-2000; Hologic, Bedford, MA, USA) was used to measure the bone mineral density (BMD) in each femur. The specimens were chosen to have a BMD of less than 0.8 g/cm^2^ because a patient is at risk of osteoporosis if the BMD is more than 1 with standard deviation (SD) below the mean gender peaks for young men (0.98 ± 0.12 g/cm^2^) and women (0.92 ± 0.10 g/cm^2^) [7, 8]. Among the six paired specimens, each bone of the pair was randomly assigned to two groups, which were either in the medial wall group (*n*_med_ = 6) or lateral wall group (*n*_lat_ = 6), ensuring an equal number of left and right specimens in each group. Because only bone pairs were used, major differences in the bone quality among the groups were excluded [9, 10]. During the preparation, instrumentation, and biomechanical testing, the specimens were intermittently sprayed with normal saline to maintain hydration.

This study was approved by the Ethics Committee of Dayi Hospital of Shanxi Medical University, and participants’ next of kin provided informed consent before commencing the present study.

### Specimen preparation

**Fig. 1:**
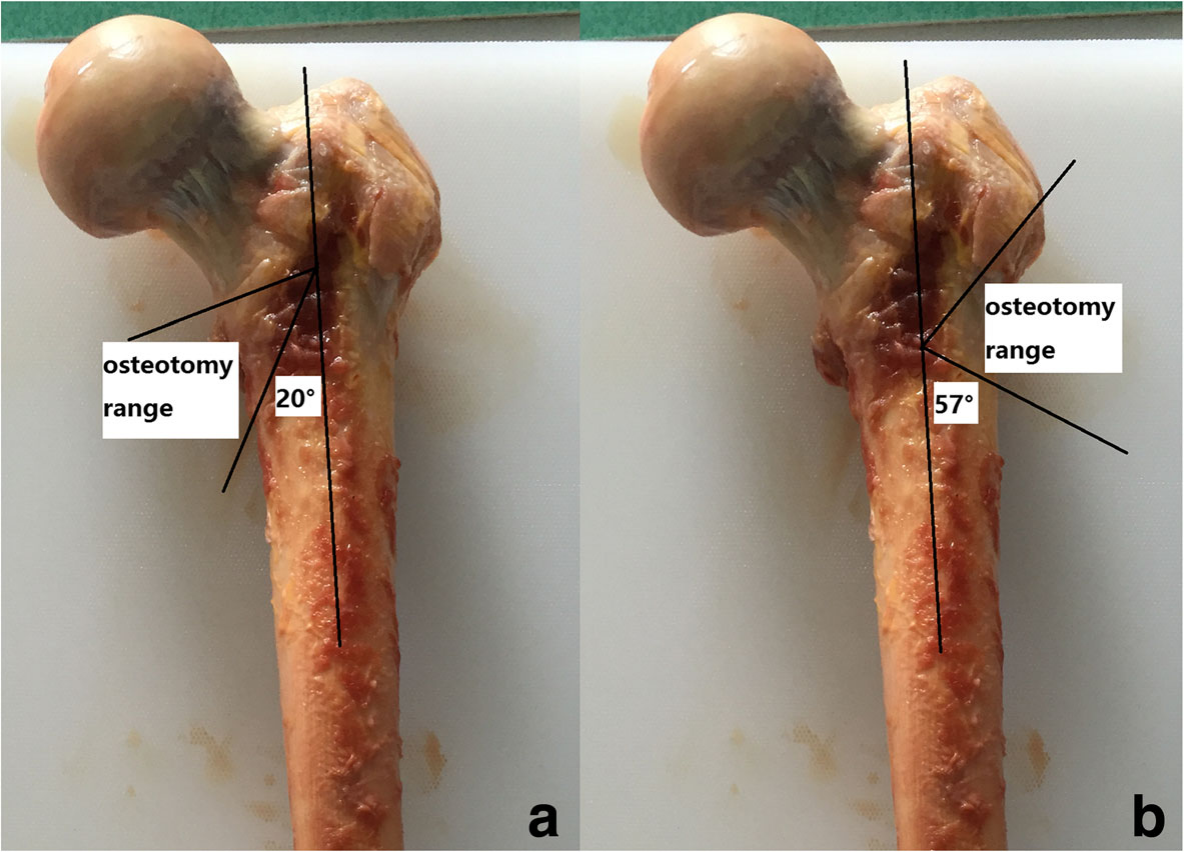
Illustration of the osteotomy range for the medial wall (**a**) and the lateral wall (**b**) of intertrochanteric region

Before the testing, all specimens were thawed at room temperature. Surrounding soft tissues were removed from each femur. Thereafter, an oscillating saw was used to create a medial wall defect model in the femoral intertrochanteric region simulated from Arbeitsgemeinschaft für Osteosynthesefragen/Orthopedic Trauma Association (AO/OTA) classification type 31A2 [8, 11] for the medial wall group. Similarly, a lateral wall defect model was created for the lateral wall group simulated from AO/OTA classification type 31A3.1 [12, 13]. For the medial wall defect model, the first osteotomy line was made at an angle of 20° with respect to the axis of the femur. It started from the bottom of the lesser trochanter, passed superolaterally through the intertrochanteric ridge, and terminated at the middle of the diaphysis. The other line was created from the top of the lesser trochanter to the middle of the diaphysis and terminated at the intersection of the first osteotomy line. The wedge of the medial wall was removed (Fig. 1a). With regard to the lateral wall defect model, the first osteotomy line was made at an angle of 57° with respect to the axis of the femur. It started at the bottom level of the lesser trochanter superomedially run and terminated at the middle of the diaphysis. The other line was created from the top of the lateral wall [4] and terminated at the intersection of the first osteotomy line. The wedge of the lateral wall was removed (Fig. 1b).

After the distal part of the femur was cut off at a 30-cm distance from the tip of the greater trochanter, the femora were placed in steel cylinders and embedded in ethoxyline resin (E-44, Tervan, Feicheng, China) at a height of 10 cm. To imitate a physiological inclination during a single-leg stance, the proximal femora were tilted at 15° of adduction, 0° of flexion, and 0° of internal rotation using a standardized positioning device [14, 15].

**Fig. 2:**
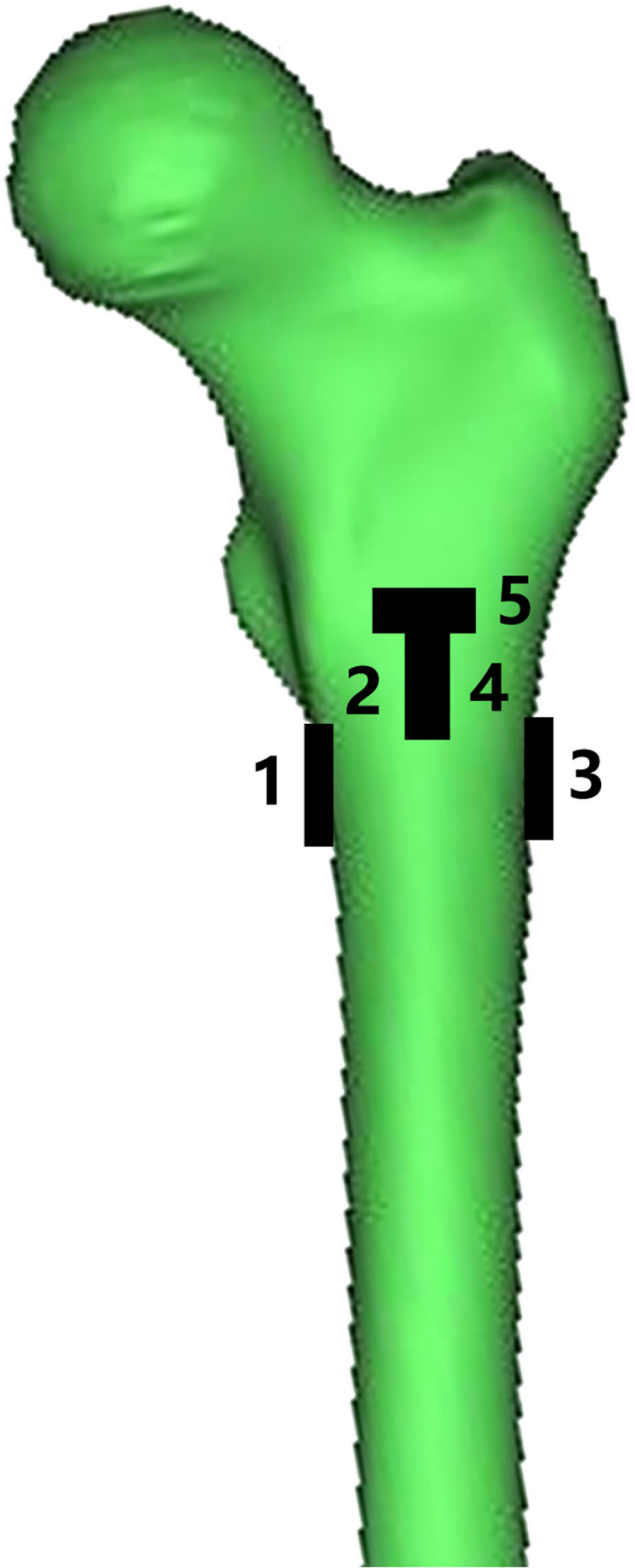
Diagram showing the positioning of the uniaxial strain gauges

Five uniaxial strain gauges (TST120-5AA, Test Electron, Jinjiang, China) were bonded to the femoral intertrochanteric region to measure the strain. For each femur pair, the position of gauge 1 was medially applied below the bottom of the lesser trochanter. Gauge 2 was anteriorly bonded below the intersection of the osteotomy line. Gauges 3 and 4 were laterally and posteriorly installed, corresponding to the levels of gauges 1 and 2, respectively. Gauge 5 was proximally bonded beside gauge 2 (Fig. 2). The detached part of each femur was provided with a separate strain gauge, which served as a resistance for temperature compensation of the room temperature.

Before the gauges were installed, the bone surface was smoothened using a sandpaper, degreased with acetone, and dried in an O_2_ stream. In addition to gauge 5 being bonded perpendicular to the diaphyseal axis of the femur, the other four gauges were installed in parallel. After exact localization, cyanoacrylate adhesive was used to cement the uniaxial strain gauges. The leads of the gauges were soldered to wires connected to a strain tester (TST3826F-L, Test Electron, Jinjiang, China). The electrical continuity, insulation, and internal resistance (120 Ω) were carefully checked for all connections, as recommended by the manufacturer. The accuracy (1%) of the strain gauges relied on the manufacturer calibration.

### Biomechanical loading and instrumentation

**Fig. 3:**
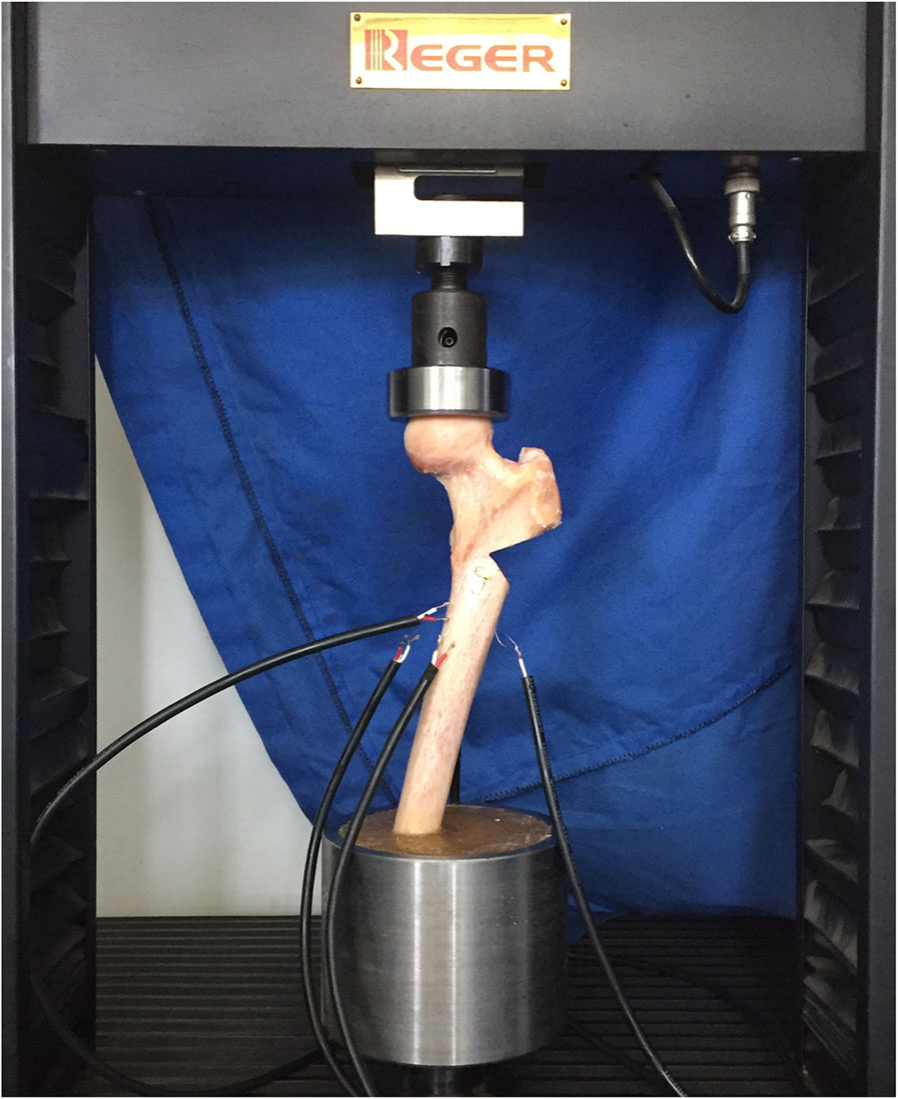
Illustration of the test setup with a custom-made spherical cap

Vertical loads were applied to the femora using a servohydraulic testing machine (RGT-20A, 20 KN nominal force, Reger Instrument, Shenzhen, China). The specimens, together with the steel cylinders, were mounted in the testing machine using a customized steel bolt. A custom-made spherical cap, which simulated the shape of the acetabulum, was fastened to the load cell and used to achieve equal load distribution on the femoral head (Fig. 3).

For the axial loading test, the preload was circulated three times under the same velocity (10 mm/min) and the same maximum load (100 N) before the official test to stabilize the construct. Prior to the official testing, the position of the load cell after preloading was manually adjusted to ensure that the spherical cap was in contact with the femoral head, and the compression load of the load cell was less than 1 N. This position of the load cell was set as the baseline to record the femoral axial displacement. Meanwhile, the strain gauges were adjusted to zero. The specimen was then loaded in a compression state at a loading rate of 5 mm/min from the baseline position. The testing was stopped when the specimen failed. Failure was defined as the appearance of a new fracture in the femur or when an acute change in the load-displacement curve occurred [13].

After the specimen failed, a PFNA with a helical blade (Double Medical, Xiamen, China) was then implanted following the surgical technique recommended by the manufacturer. To ensure consistency, all preparations and implantation procedures were performed by the same experienced orthopedic surgeons. An archiater supervised the “surgeries” and inspected the bone-implant constructs to ensure that the implants were properly installed.

The second axial loading test for the implant-femur constructs was performed using the same protocol as that of the first loading after the instrumentation. Failure was defined as a fracture in the femoral neck or shaft, and/or cutout/cut-through, and/or implant failure, and/or sudden drop in the load resistance observed in the load-displacement curve [9], or an axial displacement of the actuator of more than 20 mm [8].

For the axial testing, a load-displacement curve was plotted for each specimen, and the stiffness was calculated as the slope of the linear portion of the curve. The strains in each specimen were recorded at load levels of 350, 700, and 1800 N and the failure load.

### Statistical analysis

After testing for normality (Shapiro-Wilk test), paired *t* test or Wilcoxon signed-rank test was performed to assess the differences between the groups in terms of the investigated variables (demographics, axial stiffness, axial displacement, and strain). The data were evaluated using the SPSS version 21 statistics software. The results are presented as mean and SD. *p* < 0.05 was used as the cutoff for significance.

## Results

The mean age of the six donors was 77.17 ± 4.36 years (range 70–82 years). With respect to the bone geometry data, no difference existed between the two groups. In addition, no significant difference in the BMD existed between the medial wall and lateral wall groups (*p* = 0.463) (Table 1).

**Table 1:** Comparison of measured parameters of femora in each group (mean ± SD)

Group	Medial wall	Lateral wall	*p*
BMD (g/cm^2^)	0.506 ± 0.057	0.508 ± 0.144	0.463
Head diameter (mm)	47.60 ± 1.92	47.43 ± 2.21	0.687
Neck length (mm)	45.32 ± 1.34	44.99 ± 1.35	0.381
Neck-shaft angle (°)	136.91 ± 6.24	136.68 ± 5.28	0.653
Anteversion angle (°)	12.00 ± 2.28	12.83 ± 2.32	0.093

With regard to the failure modes, in the medial wall group, five specimens were superolaterally fractured from the intersection to the greater trochanter along the intertrochanteric ridge, and one specimen was horizontally fractured to the bottom of the greater trochanter. Meanwhile, in the lateral wall group, all specimens medially fractured from the intersection to the lesser trochanter. After PFNA implantation, all constructs failed due to the greater trochanter and/or new lateral wall fractures in the medial wall group. In the lateral wall group, five constructs failed due to the greater trochanter and/or new lateral wall fractures and one failed due to the new medial wall fracture.

**Fig. 4:**
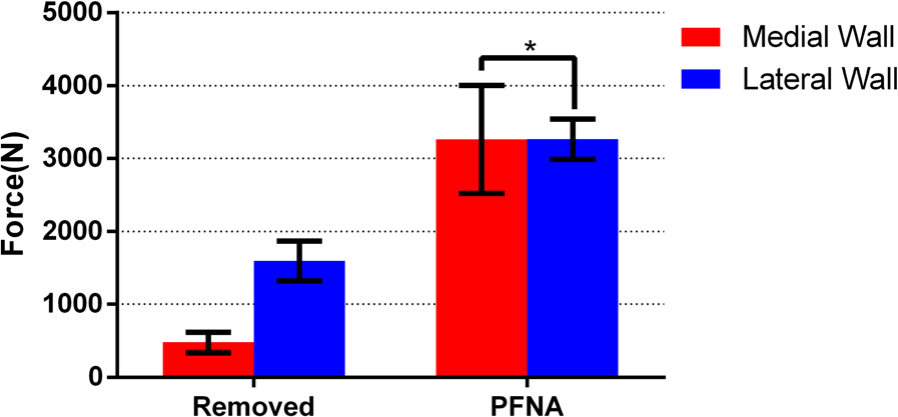
Failure loads for all tested femora in different groups (*p = 0.990)

The failure loads for all defect model specimens showed a significant difference between the two groups (*p* < 0.001). However, the difference disappeared after the PFNA implantation (*p* = 0.990). Regardless of the medial or lateral wall group, the failure loads of the PFNA-stabilized femora were significantly greater than those in the defect model femora (*p* < 0.001) (Table 2, Fig. 4).

**Table 2:** Results of the mechanical test series at failure point (mean ± SD)

	Medial wall	Lateral wall	*p*
Failure load (N)			
Removed	476.05 ± 138.85	1596.78 ± 273.17	< 0.001
PFNA	3262.63 ± 742.73	3267.66 ± 276.79	0.990
*p*	< 0.001	< 0.001	
Axial stiffness (N/mm)			
Removed	225.33 ± 36.31	911.43 ± 158.31	< 0.001
PFNA	550.71 ± 55.89	787.69 ± 84.11	0.001
*p*	< 0.001	0.020	
Displacement (mm)			
Removed	4.37 ± 0.69	3.23 ± 0.96	0.050
PFNA	6.51 ± 1.04	7.15 ± 1.27	0.491
*p*	< 0.001	< 0.001

**Fig. 5:**
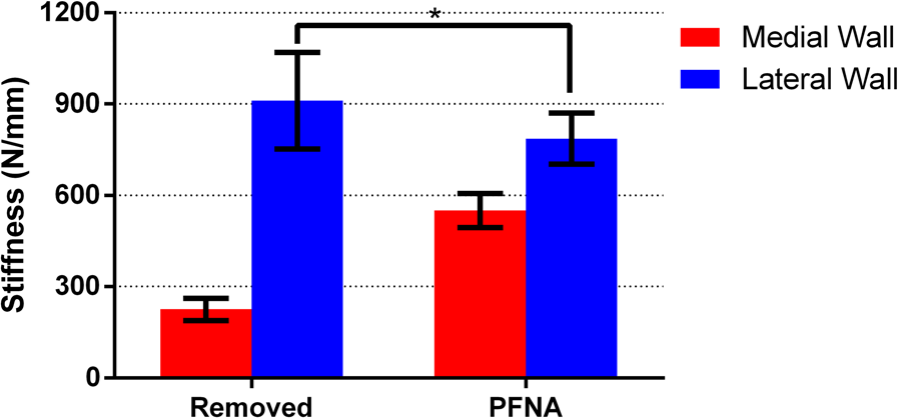
Axial stiffness for all tested femora in different groups. Statistically significant reduction of the stiffness of PFNA implanted femora compared with the defect modeling specimens in the lateral wall group (*p = 0.002)

The axial stiffness of the lateral wall-removed femora was significantly higher than that of the medial wall-removed specimens (*p* < 0.001). After the PFNA implantation, the axial stiffness of the lateral wall group remained higher than that of the medial wall group (*p* = 0.001). However, the axial stiffness of the lateral wall group showed that the axial stiffness of the lateral wall-removed femora was higher than that of the PFNA-stabilized femora (*p* = 0.020). This result was in contrast to that of the medial wall group (*p* < 0.001) (Table 2, Fig. 5).

**Fig. 6:**
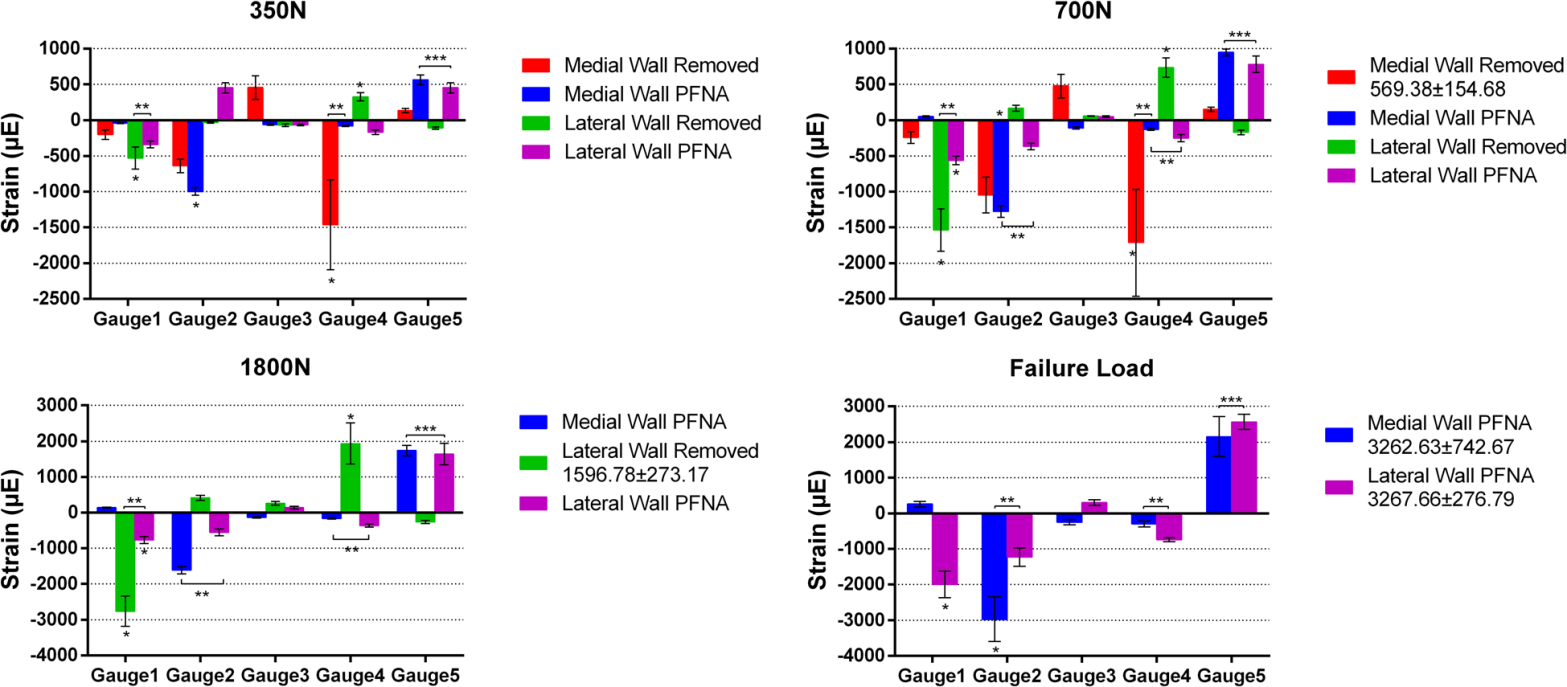
Bar chart showing the strain values of all tested femora in different groups at different load level (*showing the part of strain concentration, **p < 0.05, ***p > 0.05)

The results of the strains for all specimens in the biomechanical load series at 350, 700, and 1800 N and at the failure load are listed in Table 3. In the medial wall group, the strains in the intertrochanteric region for all defect model femora were mainly concentrated on the posterior wall (compressive strain) under an axial load. After the PFNA implantation, the strain values in the posterior wall were significantly lower than those in the defect model femora (*p* < 0.05), and the strains were mainly concentrated on the anterior wall (compressive strain). Moreover, for the lateral wall group, the strains in the intertrochanteric region for all defect model femora were mainly concentrated on the medial (compressive strain) and posterior (tensile strain) walls under an axial load. After the PFNA implantation, the strain values in the medial and posterior walls were significantly lower than those in the defect model femora (*p* < 0.05). However, the strain pattern in the posterior wall had changed to a compressive strain. Furthermore, the strains in the intertrochanteric region remained concentrated on the medial wall (compressive strain) (Table 3, Fig. 6).

**Table 3:** Results of the strain value for intertrochanteric region (mean ± SD)

Load level		Medial wall		Lateral wall	
		Removed	PFNA	Removed	PFNA
350 N	Gauge 1	− 200.17 ± 67.24	− 39.67 ± 6.62	− 529.33 ± 154.42	− 339.00 ± 47.48
	Gauge 2	− 639.00 ± 93.94	− 998.50 ± 53.55	− 31.50 ± 6.98	451.67 ± 68.01
	Gauge 3	456.00 ± 164.06	− 61.17 ± 7.36	− 67.17 ± 18.76	− 64.17 ± 10.05
	Gauge 4	− 1464.17 ± 627.05	− 77.83 ± 9.77	330.33 ± 58.86	− 167.00 ± 34.17
	Gauge 5	138.83 ± 29.73	562.83 ± 68.92	− 108.33 ± 15.68	451.67 ± 68.01
700 N	Gauge 1	NA	54.33 ± 9.31	− 1539.00 ± 298.08	− 561.83 ± 60.34
	Gauge 2	NA	− 1279.17 ± 82.85	169.33 ± 41.31	− 366.33 ± 47.07
	Gauge 3	NA	− 106.17 ± 13.96	58.67 ± 2.81	51.33 ± 9.81
	Gauge 4	NA	− 126.67 ± 13.78	734.50 ± 136.55	− 249.17 ± 49.37
	Gauge 5	NA	947.67 ± 47.83	− 168.67 ± 33.32	781.00 ± 115.90
1800 N	Gauge 1	NA	140.83 ± 15.75	NA	− 770.00 ± 98.26
	Gauge 2	NA	− 1618.0 ± 105.73	NA	− 554.83 ± 91.74
	Gauge 3	NA	− 137.50 ± 16.72	NA	138.50 ± 43.43
	Gauge 4	NA	− 161.83 ± 19.09	NA	− 368.00 ± 44.41
	Gauge 5	NA	1742.83 ± 143.35	NA	1643.83 ± 302.79
Failure load	Gauge 1	− 241.61 ± 81.12	258.67 ± 77.06	− 2761.50 ± 422.05	− 2002.83 ± 373.60
	Gauge 2	− 1048.33 ± 252.15	− 2973.83 ± 620.60	413.83 ± 68.04	− 1233.00 ± 257.59
	Gauge 3	478.17 ± 162.90	− 250.33 ± 72.50	260.67 ± 54.47	303.33 ± 82.93
	Gauge 4	− 1715.26 ± 747.24	− 299.17 ± 81.17	1937.50 ± 571.17	− 742.17 ± 60.50
	Gauge 5	155.07 ± 28.48	2155.67 ± 556.98	− 264.50 ± 45.33	2560.17 ± 212.64

Irrespective of the medial or the lateral wall group, the horizontal strain of the anterior wall in the PFNA-stabilized femora was higher than that in the defect model femora, and no significant difference was found between the medial and lateral wall groups (*p* = 0.182). However, for the axial strain in the anterior wall after the PFNA implantation, the strain in the lateral wall group was significantly lower than that in the medial wall group (*p* = 0.003). Nevertheless, for the axial strain on the posterior wall after the PFNA implantation, the result is opposite to that on the anterior wall. The strain in the medial wall group was significantly lower than that in the lateral group (*p* < 0.001) (Table 3, Fig. 6).

## Discussion

This study compared the biomechanical effect between the medial and lateral walls in the femoral intertrochanteric region of human cadaver femora. In our study, we used only bone pairs for comparison and randomly assigned them to the medial or lateral group with an equal number at the left and right sides of the femora. The use of contralateral femora as an appropriate control for each group was proven by a previous study [10]. Our current study showed that no statistically significant difference existed with regard to the morphological characteristics and the BMDs among the femora in the different groups.

The defect model of the medial and lateral walls in our study was simulated from AO/OTA classification types 31A2 and 31A3.1, respectively. In contrast to the previous methods of femoral defect modeling [11, 12], the present study simply removed only the medial or lateral wall and did not create a complete fracture. We believe that higher accuracy can be obtained by exploring the comparison of the force condition between the two groups.

All specimens in our study were tested in a servohydraulic testing machine tilted at 15° of adduction, 0° of flexion, and 0° of internal rotation to represent one-leg stance loading, with moments on all planes [15]. We used this established and simplified experimental setup and neglected the other influence on the proximal femur, such as the abductor strength and forces exerted by the soft tissue and other muscles in the leg, as a good compromise compared with the physiological conditions [16]. The loading on the hip depends on a number of factors such as activities and body weight [17]. Bergmann et al. studied the in vivo forces acting on the hip joint and found maximum gait loads (4 km/h) of 211–285% of the body weight and mean loads of 238% of the body weight [9, 16]. By choosing 1800 N as a comparative point, we assumed full weight-bearing in patients corresponding to a clinically relevant load to simulate a one-leg stance for the hip, which is approximately 2.6 times the average body weight [9].

With regard to the intertrochanteric femoral fractures, extramedullary plates and intramedullary nails are the two most common fixation methods. However, with respect to unstable fractures, intramedullary fixations with their shorter lever arm have a theoretical advantage over the extramedullary implants. PFNA is one of the effective intramedullary fixations developed by the AO/ASIF in 2004. The highlight of the implant is the use of a single blade with a large surface area. The blade provides an increased contact area between the bone and the implant, preventing (or at least delaying) the rotation-induced cutout, which is considered to be the most critical complication of the intramedullary nail for fixing intertrochanteric fractures [13, 18]. Many clinical studies have verified that PFNA is an effective implant in treating intertrochanteric fractures [18–20]. In our study, we used the same size of nails in each femur to ensure standardized comparison.

In this study, the failure mode of almost all defect model femora was consistent with the simulated type of intertrochanteric fractures. After the PFNA implantation, the failure mode of almost all stabilized femora was caused by the new fractures in the lateral wall, which is similar to that in the previous study [9]. The 3262- and 3267-N failure loads after the PFNA implantation corresponded to approximately 4.5 times the body weight that belonged to the medial and lateral wall groups, respectively, whereas everyday hip loads were achieved at 50–350% of the body weight [16]. In the defect model femora, the failure load in all medial wall group femora was significantly lower than that in the lateral wall group. This finding indicates that the integrity of the medial wall is a requirement for increasing the failure load. With respect to the axial stiffness, those of the lateral wall-removed femora were significantly higher than those of the medial wall-removed specimens. Moreover, in the lateral wall group, the lateral wall-removed femora were shown to be higher than those of the PFNA-stabilized femora. This finding indicates that the presence of the medial wall is a necessary condition for maintaining the stiffness of the femur.

Because the mechanical stimuli in the bone cannot be directly measured, different methods of measuring the bone surface strains have been used in a number of studies, with strain gauging as the most commonly used method [17]. The strain gauges suffer from the disadvantage of lower accuracy, providing local strain information only. As described in our study, for the axial strain on the anterior wall after the PFNA implantation, the lateral wall group strain was significantly lower than the medial group strain. Nevertheless, for the posterior wall, the medial wall group strain was significantly lower than the lateral group strain. These findings indicate that the presence of a medial wall between the anterior and posterior walls may keep the strain gap lower after the PFNA implantation and may provide a protective factor for the PFNA fixation.

Certainly, our study has some limitations. First and most important, the small sample numbers limited the statistical power of the study. Second, osteotomy was performed using a saw, produced flat bony interfaces, whereas fractures in patients usually have irregular surfaces. Third, all soft tissues and ligaments were removed to produce a standardized osteotomy, which is different from an ideal clinical situation. Fourth, we used the same size of nails to ensure a standardized comparison. However, the intramedullary stability of the implant could be reduced, especially in specimens with larger internal diameters. Fifth, we use continuous loading, rather than cyclic loading that could better simulate the physiological gait status. Sixth, we only tested the strain in the intertrochanteric region but did not test the proximal and distal strains on the femora. Last, since the specimens were extracted from cadavers, and the mean age was high in study participants, the results are not generalizable to the general population.

## Conclusion

In summary, this study demonstrated that PFNA is an effective intramedullary fixation system for treating unstable intertrochanteric fractures. Compared with the lateral wall, the presence of the medial wall in the intertrochanteric region is a necessary condition for increasing the axial failure load, maintaining the stiffness of the femur, and keeping the strain gap between the anterior and posterior walls down after the PFNA implantation. We suggest that in treating intertrochanteric femoral fractures with medial wall fractures in the elderly, the medial wall fragment should be reset and fixed as much as possible.

## Abbreviations

AO/OTA: Arbeitsgemeinschaft für Osteosynthesefragen/Orthopedic Trauma Association
BMD: Bone mineral density
PFNA: Proximal femoral nail antirotation
SD: Standard deviation
